# The association between food insecurity and mental health during the COVID-19 pandemic

**DOI:** 10.1186/s12889-021-10631-0

**Published:** 2021-03-29

**Authors:** Di Fang, Michael R. Thomsen, Rodolfo M. Nayga

**Affiliations:** 1 Department of Agricultural Economics and Agribusiness, University of Arkansas, Fayetteville, AR USA; 2 Department of Agricultural Economics and Agribusiness, University of Arkansas Division of Agriculture, Fayetteville, AR USA

## Abstract

**Objective:**

To explore the association between food insecurity and mental health outcomes among low-income Americans during the COVID-19 pandemic.

**Methods:**

We conducted a survey of 2714 low-income respondents nationwide from June 29, 2020 to July 21, 2020. A proportional odds logit model was employed to estimate the associations between food insecurity and anxiety and between food insecurity and depression.

**Results:**

Food insecurity is associated with a 257% higher risk of anxiety and a 253% higher risk of depression. Losing a job during the pandemic is associated with a 32% increase in risk for anxiety and a 27% increase in risk for depression.

**Conclusions:**

Food insecurity caused by the pandemic was associated with increased risk of mental illness. The relative risk of mental illness from being food insecure is almost three-fold that of losing a job during the pandemic. Public health measures should focus on getting direct subsidies of food purchases to poor families, especially families with children. They should also reduce the stigma and shame that is associated with accepting charitable foods.

**Supplementary Information:**

The online version contains supplementary material available at 10.1186/s12889-021-10631-0.

## Introduction

The COVID-19 pandemic has created unprecedented challenges, causing great distress on public health [1, 2] as well as the economy [3, 4]. Measures to curb the spread, i.e., quarantine, isolation, and shut down of schools and public places, have disrupted normal activities and have caused mental health problems in many people. It is not uncommon to have a mental health crisis during a public health emergency like this. For example, the severe acute respiratory syndrome (SARS) epidemic in 2003 was associated with a 30% increase in suicide among people over 65 years old and 50% of recovered patients experience anxiety [5]. Early evidence showed that depression was more than 3-fold higher during COVID-19 compared with before the pandemic in the US [6]. Given this concern, the World Health Organization warned against COVID-related mental health consequences such as loneliness, insomnia, depression, anxiety, and suicidal behavior [7].

Poor families are particularly vulnerable during this pandemic. Not only are isolation measures strongly associated with anxiety and depression [6], financial stress, loss of employment, and the burden of childcare can also contribute to mental health issues [6, 8]. During the pandemic, low income families face adverse situations related to food insecurity. Poor families often travel longer distances to acquire food and rely heavily on public transit [9], which has become limited or impossible due to the shutdown measures. With school closures, low-income families with children that rely on school meals are at a higher risk of experiencing hunger [10]. Feelings of alienation, worry, guilt, irritability and shame from being food insecure can also cause additional psychological problems [11, 12].

As a response to the pandemic, the Families First Coronavirus Act (FFCA) increased the benefit of the Supplemental Nutrition Assistance Program (SNAP) to the maximum allowable amount and enabled states to issue pandemic electronic meal-replacement benefits for households with children eligible to receive free or reduced-price school meals [13]. The Coronavirus Aid, Relief and Economic Security (CARES) Act passed an over $2 trillion economic relief package [14] that includes a one-time stimulus payment of $1200 per adult and an additional $500 for each child under 17 years old. These emergency measures have been proven to reduce economic hardship [15–17]. However, the impact of these measures on relieving the pandemic’s mental health burden has not been studied. Feelings of shame and anxiety can be intensified by the stigmatization of participation in food assistance programs and acceptance of charitable foods from venues such as food banks [18, 19].

To date, most studies regarding mental health and COVID-19 have focused on health workers [20, 21]. In this study, we explored the plausible association between food insecurity and mental health outcomes, i.e., anxiety and depression, among low-income Americans during the early months of the pandemic. We conducted a survey of 2714 low-income respondents nationwide. Our results show that food insecurity is highly associated with anxiety and depression. Remarkably, we find that the relative risk of mental illness from being food insecure is almost three-fold that of losing a job during the pandemic. This finding becomes even more critical given that our results further indicate that nutrition assistance programs, unemployment benefits, and stimulus payments are not associated with reducing the risk of mental illness. These results are robust across sub-samples of SNAP participants, respondents with or without children, age groups, and racial groups.

## Methods

### Data collection

A web-based survey was distributed to low-income adults in households at or below the 200% the federal poverty line (FPL).^1^ Informed consent to participate was obtained from participants by the survey company, Dynata.com. The survey was conducted from June 29, 2020 to July 21, 2020. *Dynata* sent out 8039 survey invitations on our behalf. The number of income-eligible individuals completing the survey was 2772, a 34.5% response rate. Some participants were excluded due to inconsistent reporting of ZIP Code, missing values on selected variables, and impossible values on SNAP, stimulus amount, and age. Our analysis sample thus consisted of 2714 individuals with complete data.

### Informed consent for survey participation

An introductory screening page informed potential respondents that the purpose of the research project was to advance knowledge of food insecurity in the USA. Only adults aged 18 years or older were asked to participate. Potential respondents were informed that participation was voluntary and that they would be allowed to terminate participation at any time during the survey. Those who provided their consent were then presented the survey questions.

### Ethical approval

In conformance with the Declarations of Helsinki, this work was approved by the Institutional Review Board (IRB) of the University of Arkansas for ethical conduct of research involving human subjects. The approved IRB protocol number is 2006268869.

### Statistical analyses

We used the well-established 10-item US Adult Food Security Survey Module by the US Department of Agriculture [22] to determine respondents’ food insecurity status. This module is considered the gold standard for measuring adult food insecurity within the US [22]. Based on responses to this survey module, there are four categories of food insecurity: (1) high food secure means all household members had access at all times to enough food for an active, healthy life; (2) marginal food secure means some members reported anxiety about food sufficiency or shortage of food in the house; (3) low food secure means at least some household members reported reduced quality, variety; and (4) very low food secure means one or more household members reported multiple indications of disrupted eating patterns and reduced food intake [23]. Affirmative responses to questions were summed to identify the four categories: high food secure (no affirmative responses), marginal food secure (1–2 affirmative responses), low food secure (3–5 affirmative responses), and very low food secure (6 or more affirmative responses). Respondents in the low and very low food secure categories are considered to be food insecure [22].

Depression was assessed using the Patient Health Questionnaire-9 (PHQ-9), a clinically validated survey to screen, measure, and diagnose the severity of depression in clinical and general populations [24, 25]. The PHQ-9 asks respondents to self-report the frequency of 9 signs for depression over the past 2 weeks, ranging from “little interest or pleasure in doing things” to “thoughts that you would be better off dead or of hurting yourself in some way.” Depression symptom categories were defined as none (0–4), mild (5–9), moderate (10–14), moderately severe (15–19), and severe (20) [24]. Anxiety was assessed using the General Anxiety Disorder-7 (GAD-7) questionnaire [26]. The GAD-7 has been successfully disseminated in adult primary care and psychiatric clinics and has been systematically evaluated in US and international samples [27]. GAD-7 total score ranges from 0 to 21. Scores of 5, 10, and 15 represent cut-points for mild, moderate, and severe anxiety, respectively.

Since both depression and anxiety outcomes are polychotomous and ordinal, a proportional odds logit model [28] was employed in the analysis. In an attempt to obtain a more robust estimate of the association between food insecurity and mental health, we included a number of important factors as covariates. These covariates included controls for SNAP participation, pandemic-related job loss, and residence in a USDA-classified food desert.^2^ The covariates also measured the receipt of additional SNAP benefits, alternative school meals (meals picked up at the school or at some other location during the pandemic-related school closures and intended to replace meals children would have otherwise received in school), foods from local and charitable sources, a stimulus check, and unemployment benefits. Finally, demographic variables such as the number of children under 18 years old, gender, age, race/ethnicity, education, marital status, household income, residential population, and household income stability were also included as covariates.^3^

We also estimated models from nine subsamples: 1) SNAP participants; 2) younger respondents (age group 18 to 39); 3) middle-age respondents (age group 40 to 59); 4) older respondents (age group 60 and older); 5) households with children; 6) households without children; 7) African American households; 8) Hispanic households; and 9) Caucasian households.

## Results

Respondent characteristics are summarized in Table 1. Food insecurity was measured annually through the Current Population Survey (CPS) Food Security Supplements and 28% of the households under 185% FPL were food insecure in 2019 [29]. Our analysis sample consisted of 2714 respondents, among which 51.6% are food insecure. Food insecurity (61.4%) is highest in the SNAP sample. The older respondents (i.e., at least 60 years old) reported the lowest level of food insecurity (28.5%). Respondents with children reported higher levels of food insecurity compared to the subsample without children. The Hispanic subsample reported higher level of food insecurity compared to the African American subsample and the White subsample.

**Table 1:**
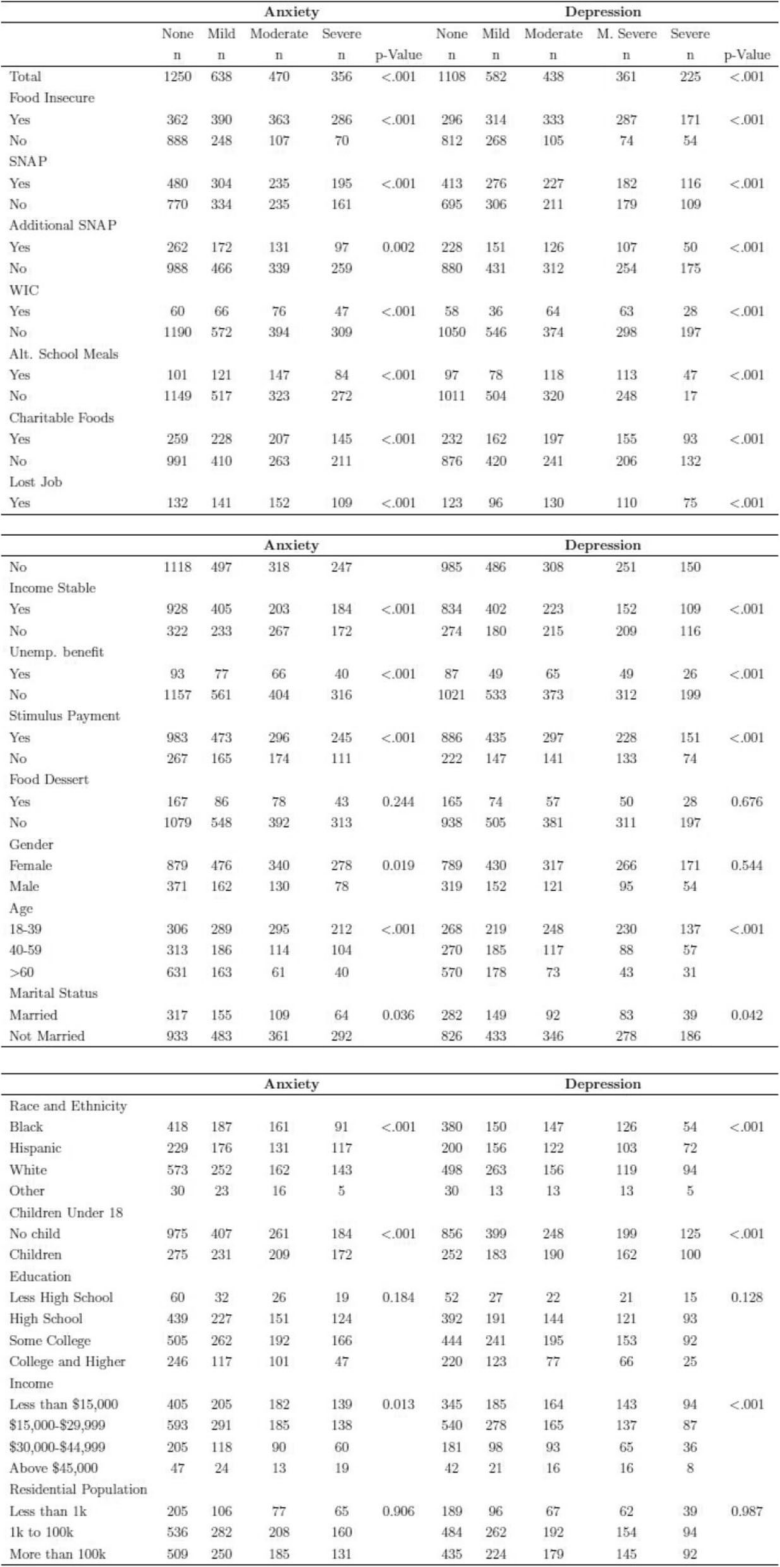
Characteristics of the sample

In the overall sample, 1250 (46.1%), 638 (23.5%), 470 (17.3%), and 356 (13.1%) showed none, mild, moderate, severe signs of anxiety, respectively; 1108 (40.8%), 582 (21.4%), 438 (16.1%), 361 (13.3%), and 225 (8.3%) showed none, mild, moderate, moderately severe, and severe signs of depression, respectively. The average anxiety score was 6.5 and the average depression score was 8.2, both in the range of mild symptoms.

Summary statistics for the subsamples are shown in Additional file 1: Table S1. Approximately 45% of the full sample reported being on SNAP (*n* = 1214), with an average monthly benefit of $241.60. Participation in the Special Supplemental Nutrition Program for Women, Infants, and Children (WIC) was 9.2 and 30.9% of respondents reported receiving charitable foods. About 19.7% (*n* = 534) reported unemployment due to the pandemic. Among those who lost jobs, 31.3% received unemployment benefits. 63.4% of our sample reported being income stable. 73.6% reported receiving a stimulus payment. Approximately 32.7% of our sample (*n* = 887) had children, with an average of 1.8 children per household. 16.7% reported receiving alternative school meals. Respondents with children also reported a much higher participation in SNAP, WIC, and school meals, consistent with the national averages [30, 31]. The average anxiety score (7.4) and depression score (9.1) is higher in the SNAP subsample, the subsample with children (anxiety: 8.5; depression: 10.2), and in the younger subsample of 18–39 years old (anxiety: 8.9; depression: 11.0). The Hispanic subsample reported higher levels of depression and anxiety compared to the African American subsample and the White subsample.

A χ2 test was performed to determine the significant differences between categories of depression and anxiety. *P* values (in Table 1) show there are significant differences between depression and anxiety in terms of food insecurity, participation of federal and local nutrition programs, and race and ethnicity. Measures indicating food-desert status, sex, education, and residential population, however, did not differ significantly by anxiety and depression, stimulus payment, unemployment benefits, pandemic-related job loss, income levels, income stability, having children, marital status, and age.

Model 1 of Table 2 exhibits the odds ratio and the 95% confidence interval (CI) obtained from the ordered logit models. Odds ratios were used as an approximation of the risk ratios of the outcomes [32]. Food insecurity is associated with a 257% higher risk of anxiety (odds ratio: 3.57; 95% CI: 3.01 to 4.23) and a 253% higher risk of depression (odds ratio: 3.53; 95% CI: 2.99 to 4.17). Loss of a job during the pandemic is associated with a 32% increase in risk for anxiety (odds ratio 1.32; 95% CI: 1.08 to 1.60) and a 27% increase in risk for depression (odds ratio: 1.27; 95% CI: 1.05 to 1.55). Income stability is associated with a 23% decrease in risk of depression (odds ratio: 0.77; 95% CI: 0.66 to 0.91). SNAP, additional SNAP benefits, WIC, and alternative school meals are not significantly associated with either anxiety or depression. Receipt of charitable foods was significantly and positively associated with anxiety (odds ratio:1.39; 95% CI: 1.17 to 1.65) and depression (odds ratio: 1.37; 95% CI: 1.16 to 1.61). This may be due to the bi-directional nature of the association due to stigma associated with visiting food pantries [11, 12]. Interestingly, receipt of unemployment benefits and receipt of a stimulus payment are not associated with mental health outcomes.

**Table 2:**
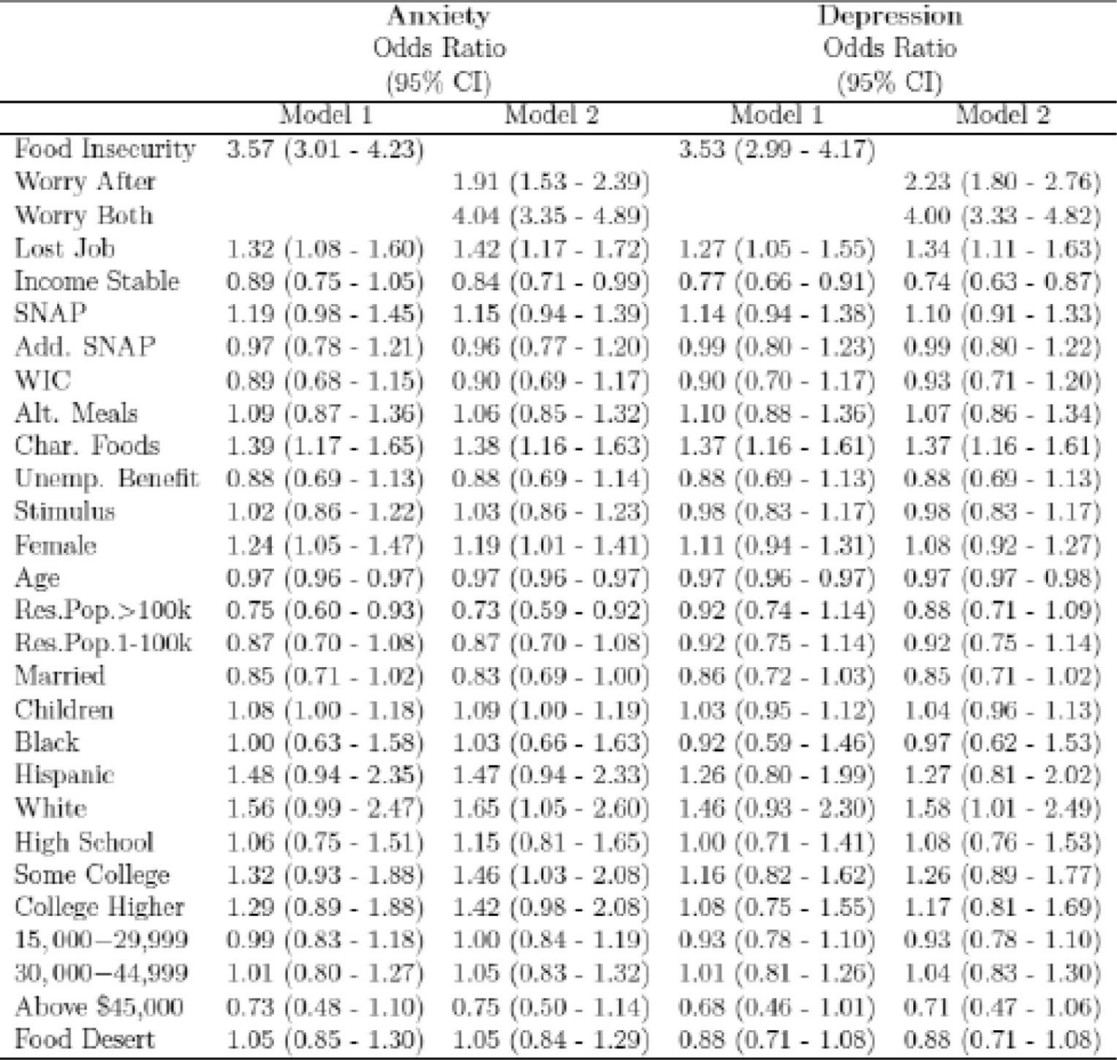
Odds ratios for anxiety and depression among 2714 low-income americans

To further assess the association between mental health and the food crisis caused by the pandemic, we also examined respondents’ degree of worry about food before and during the pandemic. We asked respondents a question about whether they were worried that food will run out before the pandemic (i.e., in January and February) and during the pandemic. Answers included” Not at all”,” Sometimes”, and” Often”. Variables were created to identify respondents who had consistently worried about food before and during the pandemic (Worry Both) from those who were only worried about food during the pandemic (Worry After). Model 2 of Table 2 shows that respondents who worried about food only after the onset of the pandemic had an odds ratio of 1.91 (95% CI:1.53 to 2.39) for anxiety and an odds ratio of 2.23 (95% CI: 1.80 to 2.76) for depression. Respondents who were worried about food before the pandemic had a much higher odds ratio of 4.04 (95% CI: 3.35 to 4.90) for anxiety and 4.00 (95% CI: 3.33 to 4.82) for depression. These estimates indicate that food hardship caused by the pandemic was associated with increased risk of mental illness for those who were newly food insecure. However, those who have experienced consistent food hardships before the pandemic had much higher risks of mental illness.

**Fig. 1:**
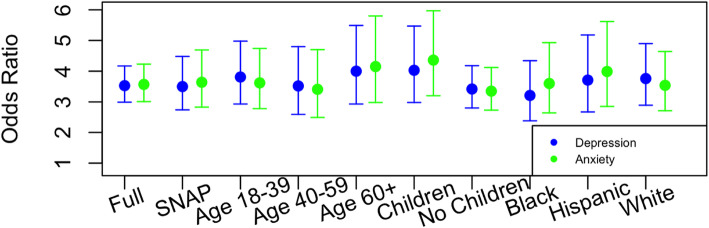
Odds ratio of food insecurity for anxiety and depression

The logit estimates for anxiety and depression across the nine sub-samples can be found in Additional file 1: Table S2 and Table S3, respectively. Respondents with children show the highest relative risk for anxiety and depression associated with being food insecure. Experiencing a pandemic-related job loss was associated with anxiety among young people between age 18 and 39, and depression among older people 60 years and older. Being income stable was not significantly associated with anxiety but is associated with lower risk of depression for people on SNAP, younger people, people with no children, and Blacks. Even though the White sample showed the lowest level of food insecurity, the relative risk of mental illnesses associated with being food insecure was higher among Whites than Blacks and Hispanics. Figure 1 shows the odds ratio (with CI) of food insecurity for anxiety and depression for all sub-samples. Consistently, food insecurity increases the relative risk of anxiety and depression in each subsample examined.

## Discussion

Food insecurity has significantly increased in the US since the start of the pandemic. Our study examined how food insecurity is associated with mental health. Food insecurity has previously been associated with mental illness in the context of developing countries [33]. Our results suggest that becoming food insecure during the pandemic is highly associated with mental health problems related to anxiety and depression. What is remarkable though is that the effect of food insecurity is three times that of the effect of losing a job during the pandemic. This study provides the first evidence of the severity of the association between food insecurity and mental health during the pandemic.

Our study also explored how economic assistance programs are associated with anxiety and depression during the pandemic. Interestingly, we found no evidence that unemployment benefits or stimulus payments decreased the relative risks of anxiety and depression. While the emergency measures by the CARES Act and the FFCA may alleviate economic hardships [17], our results show that they may not have eased the burden on mental health. Furthermore, we also did not find a significant association between mental health and nutritional programs, specifically SNAP, WIC, or alternative school meals. Charitable food sources such as food pantries are often the last resort of food procurement for poor people [18, 19]. The stigmatization of receiving food assistance might have increased anxiety and depression for those who were not food insecure before, which may help explain why food insecurity is more associated with anxiety and depression than losing a job during the pandemic. Therefore, measures should be taken to reduce the stigma and shame that is associated with accepting charitable foods [19].

There are limitations to our study. First, the 10-item US Adult Food Security Survey Module is often used to evaluate food insecurity for the past 30 days,^4^ whereas PHQ-9 and GAD-7 are used to evaluate mental health symptoms in the past 14 days. Although both food insecurity and mental illness tend to occur over time, future research should carefully validate the impact of incompatible survey windows on the sensitivity of results. Secondly, an internet-based survey has a limitation in representation due to the lack of a complicated sampling technique [34]. However, in the absence of a large-scale nationwide database on food insecurity, i.e. CPS, and mental health, our study provides timely and important insights into this issue during the pandemic. Finally, we would like to point out that our models reflect an associative, not causal, relationship between food insecurity and mental health. Future research should explore the causal relationship between these two factors with data availability and a robust identification strategy.

### Public health implications

The current pandemic has created not only a food crisis but also a mental health crisis that is associated with food insecurity. While the emergency relief measures, i.e., CARES Act and the FFCA, may have eased economic hardships, they may not have eased the burden on mental illness. Nutritional programs are created to combat food insecurity, but they are more restrictive on how their benefits are spent. For example, WIC recipients can only purchase certain approved food items. Considering the current food crisis, the magnitude of benefits from nutrition programs may also be insufficient. For example, SNAP benefits are approximately $246 per month for a household and $125 per month for a person [30]. SNAP expansion during the pandemic was only available to those who are not already receiving their maximum benefits. Therefore, even though the propensity to spend benefits from food assistance programs on food is high [35], the benefits amount itself may simply not be enough to make a difference.

Furthermore, our study highlighted the needs of families with children. School closures have forced some parents to choose between jobs and childcare. Losing access to school meals during the pandemic also added to the financial fragility of the family. Our study showed respondents with children reported worse mental health outcomes than those without children; and food insecurity was associated with the highest levels of risk for anxiety and depression among respondents with children. As US states establish guidelines for feeding children during school closures, the pandemic will intensify disparities in health for children and exacerbate the mental health issue for parents. Public health measures should focus on getting direct subsidies of food purchases to poor families, especially families with children, as well as removing the barriers to accessing charitable foods.

## Supplementary Information

**Additional file 1.**

## Data Availability

Data used in this paper collected by the authors through Dynata.com. Data used for this manuscript are maintained by the corresponding author, Professor Di Fang, and will be posted as an online supplement to the article upon acceptance and publication.

## Footnotes

1 The actual FPL cutoff depends on both income and the size of household. For example, $52,400 would place a four-person household at 200% of the FPL.

2 Food deserts are low-income census tracts with a substantial number or share of residents with low levels of access to retail outlets selling healthy and affordable foods are defined as food deserts.

3 Specifically, gender was defined as a binary variable equal to 1 for women. Age was a self-reported continuous variable. Race/ethnicity was defined as: non-Hispanic White, non-Hispanic Black, Hispanic, and other race/ethnicity. Education was defined as a categorical variable with 4 groups: less than high school graduate, high school graduate or general education diploma equivalent, some college, and college graduate or higher. Marital status was defined as a binary variable with 2 groups: married or other. Household income was defined as a categorical variable with 4 groups: $0 to $14,999, $15,000 to $29,999, $30, 000 to $44,999, and $45,000 or more. Household income stability was defined as a binary variable. Residential population was defined as a categorical variable with 3 groups: small town with less than 1000 inhabitants, city with between 1000 and 100,000 inhabitants, and city with more than 100,000 inhabitants.

4 It can also be adapted for evaluate food insecurity for a 12-month period [22]. In our case, we adapted the questions to reflect food insecurity for the past 3 months, the period between the starting of the pandemic and survey time.
