# Supplementary material for: The association between food insecurity and mental health during the COVID-19 pandemic
Source: BMC Public Health. 2021 Mar 29;21:607. doi: 10.1186/s12889-021-10631-0 (PMC8006138; doi:10.1186/s12889-021-10631-0)
Supplement: Supplementary file 1 — Additional file 1. [file 12889_2021_10631_MOESM1_ESM.pdf]

The Association Between Food Insecurity and Mental Health during the COVID-19 Pandemic

Di Fang, Michael R. Thomsen, Rodolfo M. Nayga, Jr.

supplementary file

Table S1: Summary Statistics

	Full		SNAP		18-39		40-59		60+		Children		No Child		Black		Hisp.		White	
	2714		1214		1102		717		895		887		1827		857		653		1130	
	m.	s.d.	m.	s.d.	m.	s.d.	m.	s.d.	m.	s.d.	m.	s.d.	m.	s.d.	m.	s.d.	m.	s.d.	m.	s.d.
Depression	8.2	7.3	9.1	7.2	11.0	7.3	8.2	7.1	4.7	5.7	10.2	7.4	7.2	7.0	7.8	7.2	9.6	7.3	7.6	7.2
Anxiety	6.5	6.2	7.4	6.4	8.9	6.2	6.8	6.2	3.4	4.7	8.5	6.4	5.6	5.9	6.1	6.1	7.9	6.3	6.1	6.3
Food Insecure	0.5	0.5	0.6	0.5	0.7	0.5	0.6	0.5	0.3	0.5	0.7	0.5	0.4	0.5	0.5	0.5	0.6	0.5	0.5	0.5
Lost Job	0.2	0.4	0.2	0.4	0.3	0.5	0.2	0.4	0.1	0.2	0.3	0.5	0.1	0.4	0.2	0.4	0.3	0.4	0.1	0.3
Income Stable	0.6	0.5	0.6	0.5	0.4	0.5	0.6	0.5	0.9	0.3	0.5	0.5	0.7	0.5	0.6	0.5	0.5	0.5	0.7	0.4
SNAP	0.4	0.5	1.0	0.0	0.5	0.5	0.5	0.5	0.4	0.5	0.6	0.5	0.4	0.5	0.5	0.5	0.4	0.5	0.4	0.5
Add. SNAP	0.2	0.4	0.5	0.5	0.3	0.4	0.3	0.4	0.2	0.4	0.3	0.5	0.2	0.4	0.3	0.5	0.2	0.4	0.2	0.4
Add. SNAP Amt.	0.0	0.1	0.1	0.2	0.1	0.1	0.0	0.1	0.0	0.1	0.1	0.2	0.0	0.1	0.1	0.1	0.0	0.1	0.0	0.1
WIC	0.1	0.3	0.1	0.4	0.2	0.4	0.0	0.2	0.0	0.1	0.2	0.4	0.0	0.2	0.1	0.3	0.1	0.3	0.1	0.2
Alt. Meals	0.2	0.4	0.2	0.4	0.3	0.5	0.1	0.3	0.0	0.2	0.4	0.5	0.0	0.2	0.2	0.4	0.2	0.4	0.1	0.3
Char. Food	0.3	0.5	0.4	0.5	0.4	0.5	0.3	0.5	0.2	0.4	0.4	0.5	0.3	0.4	0.4	0.5	0.3	0.5	0.2	0.4
Unemp. Benefit	0.1	0.3	0.1	0.3	0.2	0.4	0.1	0.3	0.0	0.2	0.1	0.3	0.1	0.3	0.1	0.3	0.1	0.4	0.1	0.2

Table S1: Summary Statistics

	Full		SNAP		18-39		40-59		60+		Children		No Child		Black		Hisp.		White	
Stimulus	0.7	0.4	0.8	0.4	0.6	0.5	0.7	0.4	0.9	0.3	0.7	0.5	0.7	0.4	0.7	0.5	0.7	0.5	0.8	0.4
Female	0.7	0.4	0.7	0.4	0.7	0.4	0.7	0.4	0.7	0.4	0.8	0.4	0.7	0.5	0.8	0.4	0.7	0.4	0.7	0.5
Age	46.8	18.6	45.2	17.4	27.3	6.3	50.0	6.2	68.3	5.7	36.2	13.4	51.9	18.6	44.1	18.5	37.9	15.9	54.4	17.3
Pop.<1k	0.2	0.4	0.2	0.4	0.1	0.3	0.2	0.4	0.2	0.4	0.1	0.4	0.2	0.4	0.1	0.3	0.1	0.3	0.2	0.4
Pop.1-100k	0.4	0.5	0.4	0.5	0.4	0.5	0.4	0.5	0.4	0.5	0.5	0.5	0.4	0.5	0.4	0.5	0.4	0.5	0.5	0.5
Pop.>100k	0.4	0.5	0.4	0.5	0.4	0.5	0.4	0.5	0.4	0.5	0.4	0.5	0.4	0.5	0.5	0.5	0.5	0.5	0.3	0.5
Married	0.2	0.4	0.2	0.4	0.2	0.4	0.3	0.4	0.2	0.4	0.3	0.5	0.2	0.4	0.2	0.4	0.3	0.4	0.3	0.4
Num. Children	0.6	1.0	0.8	1.1	1.0	1.2	0.6	1.0	0.1	0.5	1.8	1.0	0.0	0.0	0.6	1.0	0.8	1.1	0.5	0.9
Black	0.3	0.5	0.4	0.5	0.3	0.5	0.3	0.5	0.3	0.4	0.3	0.5	0.3	0.5	1.0	0.0	0.0	0.0	0.0	0.0
Hispanic	0.2	0.4	0.2	0.4	0.4	0.5	0.2	0.4	0.1	0.3	0.3	0.5	0.2	0.4	0.0	0.0	1.0	0.0	0.0	0.0
White	0.4	0.5	0.4	0.5	0.3	0.4	0.4	0.5	0.6	0.5	0.3	0.5	0.5	0.5	0.0	0.0	0.0	0.0	1.0	0.0
Less High	0.1	0.2	0.1	0.3	0.1	0.2	0.1	0.2	0.0	0.2	0.1	0.2	0.0	0.2	0.1	0.2	0.1	0.2	0.0	0.2
High School	0.3	0.5	0.3	0.5	0.4	0.5	0.3	0.5	0.3	0.5	0.4	0.5	0.3	0.5	0.3	0.5	0.3	0.5	0.4	0.5
Some College	0.4	0.5	0.4	0.5	0.4	0.5	0.4	0.5	0.4	0.5	0.4	0.5	0.4	0.5	0.4	0.5	0.5	0.5	0.4	0.5
College Higher	0.2	0.4	0.2	0.4	0.2	0.4	0.2	0.4	0.2	0.4	0.2	0.4	0.2	0.4	0.2	0.4	0.2	0.4	0.2	0.4
<$15,000	0.3	0.5	0.4	0.5	0.4	0.5	0.4	0.5	0.3	0.4	0.3	0.4	0.4	0.5	0.4	0.5	0.3	0.5	0.3	0.5

Table S1: Summary Statistics

	Full		SNAP		18-39		40-59		60+		Children		No Child		Black		Hisp.		White	
$15k-$29,999	0.4	0.5	0.4	0.5	0.4	0.5	0.4	0.5	0.6	0.5	0.4	0.5	0.5	0.5	0.4	0.5	0.4	0.5	0.5	0.5
$30k-$44,999	0.2	0.4	0.1	0.3	0.2	0.4	0.1	0.3	0.2	0.4	0.3	0.4	0.1	0.3	0.1	0.3	0.2	0.4	0.2	0.4
>$45,000	0.0	0.2	0.0	0.2	0.1	0.2	0.0	0.2	0.0	0.1	0.1	0.3	0.0	0.1	0.0	0.2	0.0	0.2	0.0	0.2
Food Dessert	0.1	0.3	0.1	0.3	0.1	0.3	0.1	0.4	0.1	0.3	0.2	0.4	0.1	0.3	0.2	0.4	0.1	0.3	0.1	0.3

Table S2: Odds Ratio for Anxiety by Subsample

	Dependent variable: Anxiety								
	SNAP	age18-39	age40-59	age60+	children	no-children	black	hispanic	white
	(1)	(2)	(3)	(4)	(5)	(6)	(7)	(8)	(9)
Food Insecure	3.64***	3.62***	3.41***	4.15***	4.36***	3.35***	3.60***	3.99***	3.54***
Lost Job	1.25	1.30*	1.43	1.53	1.22	1.42*	1.21	1.23	1.56*
Income Stable	0.89	0.87	0.98	0.77	0.93	0.86	0.80	0.94	1.08
SNAP		1.24	1.30	0.73	1.06	1.19	1.35	1.10	1.14
Add.SNAP	1.01	0.81	1.24	1.22	0.99	0.98	1.17	0.90	0.86
WIC	0.94	0.98	0.99	2.81	1.11	0.56*	1.08	0.68	0.71
Alt. Meals	1.08	1.15	1.41	0.47	1.21	1.04	1.04	1.10	0.99
Chari. Foods	1.15	1.38*	1.22	1.69**	1.20	1.56***	1.39*	1.71**	1.25
Unemp. Benefit	0.92	0.89	0.84	1.21	1.09	0.76	0.90	0.92	0.71
Stimulus	1.00	1.05	0.95	0.86	0.81	1.20	0.89	1.18	1.08
Female	1.24	1.32*	1.32	1.03	1.42*	1.19	1.08	1.76**	1.09
Age	0.96***				0.97***	0.97***	0.97***	0.97***	0.96***
Pop.> 100k	0.73	0.73	0.77	0.65	0.53**	0.85	0.77	0.53*	0.77
Pop.1k-100k	0.84	0.81	0.68	1.27	0.59**	1.02	0.79	0.67	0.99
Married	0.87	0.70*	0.89	0.84	0.84	0.90	0.78	0.94	0.88
Children	1.03	1.09	1.02	1.28			1.14	1.04	1.10
Black	1.06	1.14	0.88	0.55	1.47	0.78			
Hispanic	1.33	1.40	1.95	0.81	2.33*	1.12			
White	1.46	1.38	1.54	0.92	2.36*	1.21			
High School	1.25	1.41	1.12	0.51	0.78	1.19	1.06	1.39	0.91
Some College	1.51	1.98*	1.31	0.58	1.08	1.38	1.28	2.20*	0.96
College Higher	1.22	1.47	1.36	0.77	0.93	1.43	1.09	1.66	1.24
15k-29,999	0.87	0.85	0.95	1.27	1.12	0.94	1.03	0.80	1.03
30k-44,999	0.79	0.94	0.90	1.44	1.06	1.02	1.12	1.03	0.87
>45k	0.89	0.91	0.47	0.14	0.66	1.48	1.28	0.49	0.54
Food Dessert	1.01	1.18	1.01	0.91	0.88	1.19	1.11	1.18	0.94
Observations	1,210	1,101	712	893	883	1,823	856	648	1,128

Note: *p<0.05; **p<0.01; ***p<0.001

Table S3: Odds Ratio for Depression by Subsample

	Dependent variable: Depression								
	SNAP	age18-39	age40-59	age60+	children	no-children	black	hispanic	white
	(1)	(2)	(3)	(4)	(5)	(6)	(7)	(8)	(9)
Food Insecure	3.50***	3.81***	3.52***	4.00***	4.03***	3.42***	3.21***	3.71***	3.76***
Lost Job	1.30	1.18	1.39	1.95*	1.28	1.30	1.30	1.20	1.36
Income Stable	0.70**	0.72**	0.86	0.76	0.80	0.75**	0.74*	0.77	0.89
SNAP		1.27	1.10	0.93	1.10	1.09	1.19	1.21	1.03
Add.SNAP	1.02	0.82	1.16	1.17	0.92	1.07	1.24	0.72	1.01
WIC	0.97	0.97	1.01	1.91	1.04	0.61	1.15	0.61*	0.90
Alt. Meals	1.07	1.20	1.47	0.22*	1.12	1.35	1.14	1.25	0.95
Chari. Foods	1.22	1.31*	1.18	1.77**	1.11	1.60***	1.20	1.50*	1.53**
Unemp. Benefit	0.76	0.98	0.82	0.83	1.16	0.73	1.05	0.85	0.69
Stimulus	1.08	1.00	0.91	0.92	0.83	1.11	0.88	1.20	0.93
Female	1.01	1.21	1.30	0.85	1.34	1.05	0.84	1.65**	1.06
Age	0.96***				0.97***	0.97***	0.97***	0.97***	0.97***
Pop.> 100k	0.80	0.78	0.97	0.99	0.67*	1.06	0.78	0.90	0.97
Pop.1k-100k	0.85	0.77	0.81	1.36	0.67*	1.08	0.70	1.00	1.06
Married	0.94	0.75*	0.87	0.80	0.89	0.89	0.72	0.85	0.97
Children	0.96	1.02	0.97	1.32			1.07	1.06	0.99
Black	0.84	1.03	0.63	1.27	1.33	0.70			
Hispanic	1.02	1.20	1.25	1.54	2.02*	0.89			
White	1.19	1.25	1.05	2.21	2.02*	1.14			
High School	1.19	1.17	0.88	0.88	0.92	1.07	1.26	1.26	0.82
Some College	1.44	1.30	1.06	1.05	1.12	1.18	1.39	1.49	0.92
College Higher	1.23	1.10	0.74	1.29	1.04	1.09	1.28	1.10	1.00
15,000-29,999	0.86	0.72*	0.89	1.43*	0.82	0.98	1.03	0.66*	1.01
30,000-44,999	1.00	0.91	0.95	1.55	0.88	1.13	1.13	0.93	0.98
>45,000	0.80	0.80	0.47	0.27	0.56*	1.17	1.07	0.43*	0.58
Food Dessert	0.93	0.92	0.76	0.94	0.76	0.94	0.80	1.37	0.83
Observations	1,210	1,101	712	893	883	1,823	856	648	1,128

Note: $^{*}p<0.05$; $^{**}p<0.01$; $^{***}p<0.001$
